# Building an Affordances Map With Interactive Perception

**DOI:** 10.3389/fnbot.2022.504459

**Published:** 2022-05-10

**Authors:** Léni K. Le Goff, Oussama Yaakoubi, Alexandre Coninx, Stéphane Doncieux

**Affiliations:** Sorbonne Université, CNRS, Institut des Systémes Intelligents et de Robotique, ISIR, Paris, France

**Keywords:** autonomous exploration, affordance learning, interactive perception, perceptual map, online learning

## Abstract

Robots need to understand their environment to perform their task. If it is possible to pre-program a visual scene analysis process in closed environments, robots operating in an open environment would benefit from the ability to learn it through their interaction with their environment. This ability furthermore opens the way to the acquisition of affordances maps in which the action capabilities of the robot structure its visual scene understanding. We propose an approach to build such affordances maps by relying on an interactive perception approach and an online classification for a real robot equipped with two arms with 7 degrees of freedom. Our system is modular and permits to learn maps from different skills. In the proposed formalization of affordances, actions and effects are related to visual features, not objects, thus our approach does not need a prior definition of the concept of object. We have tested the approach on three action primitives and on a real PR2 robot.

## 1. Introduction

Nowadays, robots can achieve specific tasks with a high accuracy in controlled environments, such as automated factories. In such environments, the engineers can anticipate all the aspect of the problem at hand and then simply program the robot to achieve its goal. However, in open and dynamic environments, it is difficult to anticipate everything. To solve tasks in such a context, robots need adaptive skills. A way to approach this issue is to let it explore its surrounding and learn from its experiences. By exploring its environment, the robot is able to build its own representation according to its embodiment, skills and goals.

The psychologist E. Gibson claimed that acquiring perception is “discovering distinctive features and invariant properties of things and events” (Gibson, 2000) and “discovering the information that specifies an affordance” (Gibson, 2003). This could be interpreted, for a robot, as the system must isolate regularities and invariance in the data collected during an exploration to build representations. And, these representations are affordances. The concept of affordances was introduced by Gibson (1966, 1979).

With this concept, Gibson wanted to highlight that objects have inherent “values” and “meanings” which could be perceived by an agent and could be linked to its possible actions on those objects. An animal or a human thus perceives the world through the actions it can perform according to their abilities and the elements in the environment. However, in Gibson's view, animals and humans do not need computation or representations to perceive the affordances. A robotic system needs to build a representation from its visual stream in order to learn complex task e.g., recognizing objects with the aim of interacting with them. Fortunately, psychologists continued to work on the ecological's approach and refined the concept of affordance. From these studies, we summarized several main aspects of affordances suitable for a robotic system:

- Affordances *emerge* from the relation between the agent and the environment (Chemero, 2003);
- *Functionality* is an inherent property of objects or parts of the environment. A functionality could become an affordance if the agent has some knowledge about it and if the agent is able to use it (Steedman, 2002a,b);
- Affordances are perceived thanks to an internal representation which maps the agent perceptions and actions (Vera and Simon, 1993).
- Affordances are not always self-evident. Therefore learning and exploration could be needed to perceive affordances. *Signifiers* could be built to help an agent perceive affordances (Norman, 2013).

In this article, we state that an affordance is an emergent relationship in the agent-environment system. Thus, an affordance is a relationship between a sensory signal, the agent skills and the possible effect that would result from the agent's actions. Affordances are learned from experience of the agent interacting with the real world, and as a result of this learning, affordances can then be directly perceived in the environment. Moreover, for the affordances to be learned, the environment needs to have distinctive and coherent sensory signals associated with actions and effects, in other words, they need to be discoverable. This definition is coherent with the ones proposed in the developmental robotic literature (Zech et al., 2017).

The work, presented in this article, proposed a system to learn a perceptual representation based on affordances. The aim is to answer to the following problematic: *How can a robot with a toolbox of action primitives build a representation of the environment based on affordances by autonomous exploration?* The robotic system learns from data collected during an autonomous exploration by *interacting* with the environment, following the *interactive perception* paradigm.

Interactive perception aims at learning perception from interaction. According to Bohg et al. (2017), a robotic system, with interactive perception, isolates regularities in the combined space of sensory signals, motor commands and time. This meets the vision of Gibson about learning. Therefore, it is natural to use interactive perception to let a system autonomously learn affordances.

However, most works in interactive perception are interested in learning objects representations for recognition, segmentation or manipulation. To achieve their goal, these methods need to introduce assumptions about the structure of the environment or about the objects themselves. These assumptions reduce the range of environments that the robot could face. One of our previous works (Le Goff et al., 2019) addresses this issue by proposing a method to learn a perceptual map, called *relevance map*, through interactive perception with minimum environment-specific assumptions and by using a classifier trained online called the *Collaborative Mixture Models* based on Gaussian Mixture Model. This article presents an extension of this previous work in which a relevance map is built based on data collected through the interaction of a robot with an environment through a push primitive. This approach is within the scope of interactive perception as it learns a representation of the world through interactions with an environment. This relevance map was representing the relevant areas in a visual scene for the push primitive, i.e., the areas that would produce an effect after the application of the push primitive. Thus, the relevance map represents areas which afford a certain action. In the present study, relevance maps relative to several affordances are learned: pushable objects, liftable objects, and pressable buttons. These maps are then combined to produce an affordances map. This affordances map is a starting point for further developmental steps, and provides the knowledge needed to bootstrap a decision process (Doncieux et al., 2018).

The main contribution of this work is a modular framework to learn low level affordances represented by a perceptual map. The *affordances map* gives to the robot a rich and direct perception of its surrounding according to the actions it can perform. This is close to Gibson's first conception of affordances.

To support this contribution, three series of experiments are presented in this article. In each of these experiments the robot explores its environment using interactive perception with a specific action primitive and a specific effect detector: push primitive with movement detector, lift primitive with movement detector, and push primitive with button activation detector. The goal of these experiments is to build a relevance map related to the primitive and the effect detector used.

Before explaining the method used in these experiments in Section 3, related works about affordance learning are described in Section 2. You can find a detailed description of the experimental protocol and of the results in the Sections 4 and 5.

## 2. Related Works

Affordances have raised a lot of interest in the developmental robotics community these last 10 years, as shown by the numerous reviews and surveys dedicated to this topic (Sahin et al., 2007; Horton et al., 2012; Jamone et al., 2016; Min et al., 2016; Zech et al., 2017).

According to a recent survey (Zech et al., 2017), among 146 reviewed articles, 104 articles consider learning affordances directly from a meso level, i.e., considering objects as a whole, while only 27 articles consider it from a global level, i.e., by considering the whole environment and only 15 articles from a local level. The global level considers the whole environment and thus allows the learning system to integrate the context. The context is important to predict or to do recognition of high-level affordances. Most articles on affordance use the meso level because for most actions having a complete model of an object is practical. For instance, for successful grasps, the object states such as orientation and position or shape are important information. Learning affordances at a local level allows the system to perceive them directly, Moreover considering the local level is simpler and is thus suitable to bootstrap the system.

The proposed method is based on learning affordances from local visual features, so from the local level. Therefore, this section is focused on articles interested in learning affordances at a local level. From these 15 articles, 11 are interested in linking local descriptors to the possible actions applicable in the present environment for quick or direct perception of affordances. From these articles, 6 learn from exploration using an interactive perception approach. This shows that the question of learning affordances from local features using exploration has not been extensively studied yet. This section reviews different groups of works addressing this question. A first group aims at learning several kinds of affordances with supervised learning on annotated datasets, a second one focuses on the object grasping issue and finally, works that do not fit in these two categories are mentioned.

Some studies use an annotated dataset to train a model of affordance classification and then integrate this model in a robotic framework, as a tool for planning, task solving or manipulation. Myers et al. (2015) study tool use affordances. They train a classifier on superpixels using SLIC. Achanta et al. (2010) have extended it to work on RGB-D images, with features related to shape. Two classifiers are proposed in this work. A first one is called superpixel hierarchical matching which is computationally demanding and slow for prediction. The second one is a structured random forest which achieves fast prediction and is therefore suited to real-time systems, but this last classifier is trained offline. AfRob method proposed by Varadarajan and Vincze (2012) is used to classify affordances from 2D images. It is a deep neural network trained in batch. AfRob is the adaptation of, previously proposed, AfNet, from the same authors, to robotics constraints (fast prediction, light computation). Katz et al. (2014) aim at detecting affordances from stacks of objects. With this aim, an SVM linear classifier is used to learn pulling, pushing, and grasping affordances. As they use objects with simple shapes and only consider their facets as features, i.e., small planar surfaces which compose a 3D shape, they can use a simple linear classifier, especially if trained offline on an annotated dataset. In the same idea, Kim and Sukhatme (2014) proposed a method to detect affordances of surfaces based on a geometrical analysis of the pointcloud, K-means clustering, and logistic regression.

Those methods proposed efficient tools for robotic systems to detect affordances, but they are all based on supervised learning on datasets annotated by a human expert. Annotating is a costly process that naturally limits the learned model to the datasets produced by the expert. Moreover, affordances in ecological psychology depend on the agent body structure and on the actions it is capable of. Another approach is thus to let the robot explores its environment with one or several actions and collects information about the affordances in its surrounding and discovers by itself the affordances.

A group of works (Bierbaum et al., 2009; Montesano and Lopes, 2009; Kraft et al., 2010; Krüger et al., 2011; Popović et al., 2011; Dang and Allen, 2014) are focused on building affordance maps of successful grasps on an object. Bierbaum et al. (2009) let a robotic hand with tactile sensors explore an unknown object in simulation. The robot hand has five fingers including a thumb. The system detects a potential grasp by finding opposite flat surfaces. Then, candidate areas for grasping are determined offline on the basis of the geometrical analysis of local shape features. The analysis is a heuristic based on the configuration of the hand used. Alternatively, Montesano and Lopes (2009) propose a trial and error process to determine the probability of success of a grasp on parts of an object. Learning is based on local visual features in a Bayesian framework. The robot tries to grasp several times the same object part and, with a Bernoulli-beta distribution based on the successes or failures, the system determines the probability of the graspability of this part. In the same idea, Dang and Allen (2014) proposed a system that learns a graspable affordance map on objects but they add what they call semantic constraints. These constraints are designed by a human to force grasping to be compatible with a specific task. In the same way, Popović et al. (2011) and Krüger et al. (2011) use Early Cognitive Vision (ECV, Krüger et al., 2010) for preliminary image processing to extract features with a stereo camera. The features are edges, contours, textures, and surfaces. The robot tries to grasp different objects and associates ECV's features to successful grasps. A limitation of this work is that ECV needs textured or complex objects to work properly.

These works are conceptually similar to ours: a robotic system explores an environment (here an object) with an action (here grasping) and learns to associate local visual features to successful actions. However, they assume that the system is already able to extract objects from a scene and focus on it to learn grasping. In our work, the robotic system has no notion of objects. The whole environment is considered, in order to learn relevant areas for different affordances. From these areas, object candidates could be extracted as a base for the above-mentioned methods. Thus, these works correspond to a later developmental step with respect to ours.

Uǧur et al. (2007) proposed a method for learning “traversability” affordance with a wheeled mobile robot which explores a simulated environment. The robot tries to go through different obstacles: laying down cylinders, upright cylinders, rectangular boxes, and spheres. The laying down cylinders and spheres are traversable while boxes and upright cylinders are not. The robot is equipped with a 3D sensor and collects data after each action labeled with the success of going through the objects. The sample data are extracted thanks to a simulated RGB-D camera. Then, an online SVM (Bordes et al., 2005) is trained based on the collected data. The resulting model predicts the “traversability” of objects based on local features. To drive the exploration, an uncertainty measure is computed based on the soft margin of the model decision hyperplane. Finally, they tested their method on a navigation problem, on real robots and in a realistic environment. They demonstrate, by using the model learned in simulation, that the robot is able to navigate through a room full of boxes, spherical objects and cylindrical objects like trash bins without colliding with non-traversable objects.

Kim and Sukhatme (2015), with a similar idea, seek to learn pushable objects in a simulated environment using a PR2 with an RGB-D camera. The objects are blocks the size of the robot. They are either pushable in one or two directions, or not pushable. The PR2 uses its two arms to try to push the blocks. The learning process relies on a logistic regression classifier and a Markov random field is used to smooth spatially the predictions. The robot explores then the environment and collects data by trying to push the blocks. The outcome of the framework is what they called an affordance map indicating the probability of pushability of a block. When in the work of Uǧur et al. (2007) the learning is made on continuous space, in the work of Kim and Sukhatme (2015) the environment is discretized in a grid with the cells of the size of a block, thus, the learning space is discrete. Finally, they use an exploration strategy based on uncertainty reduction to select the next block to interact with.

In a more developmental perspective, Paletta et al. (2007) proposed a framework to learn composite affordances by starting from low level affordances. Their approach is split into 3 steps: first, the robot explores its environment with a reactive behavior, like a grasp reflex, and collects visual data consisting of SIFT. Then, in a second step, basic affordances are learned with simple actions such as pushing or gripping. Finally, in the third step, the robot learns composite affordances based on a combination of the basic action used in the previous step. For instance, this combination of actions allows the robot to achieve stacking. They validate their framework with a mobile robot equipped with a stereo camera and a magnetized end-effector. In a real environment the robot tries to learn to identify objects that are liftable with its magnetized end-effector.

These works (Paletta et al., 2007; Uǧur et al., 2007; Kim and Sukhatme, 2015) are close to the work presented in this article. They gather in a single study affordance learning, online learning, exploration process, and interactive perception. The affordance map of Kim and Sukhatme (2015) is close to our relevance map by the way they both segment interesting elements for the agent, but exploration and learning were conducted in simulation only, in simple environments and setups, and only one affordance was learnt. The study proposed by Paletta et al. (2007) can learn several affordances in simulation, but it was tested in reality with only one action.

Thus, they do not address the challenge of separating local visual features in complex and realistic environment. The approach proposed in this article is based on similar principles but it allows the system to learn relevance maps relative to several affordances in more complex and realistic environments and in real world-experiments. Real environments have often rich visual features in which both classes could share similar visual features. This makes the problem of classification complex. Also, exploration in a real environment produces unexpected errors with the interactions or the detection of a possible effect. By conducting experiments in the real world, our approach is confronted to such issues.

## 3. Method

The goal of this work is, for a robot, *to learn which part of an environment affords a given effect to a specific action through an autonomous exploration*. The robot is interacting with the environment thanks to an action primitive in order to collect data. The method is tested with three affordances: pushable objects, pressable buttons and liftable objects. These affordances are respectively linked to a push primitive, a press primitive and a lift primitive.

**Figure 1:**
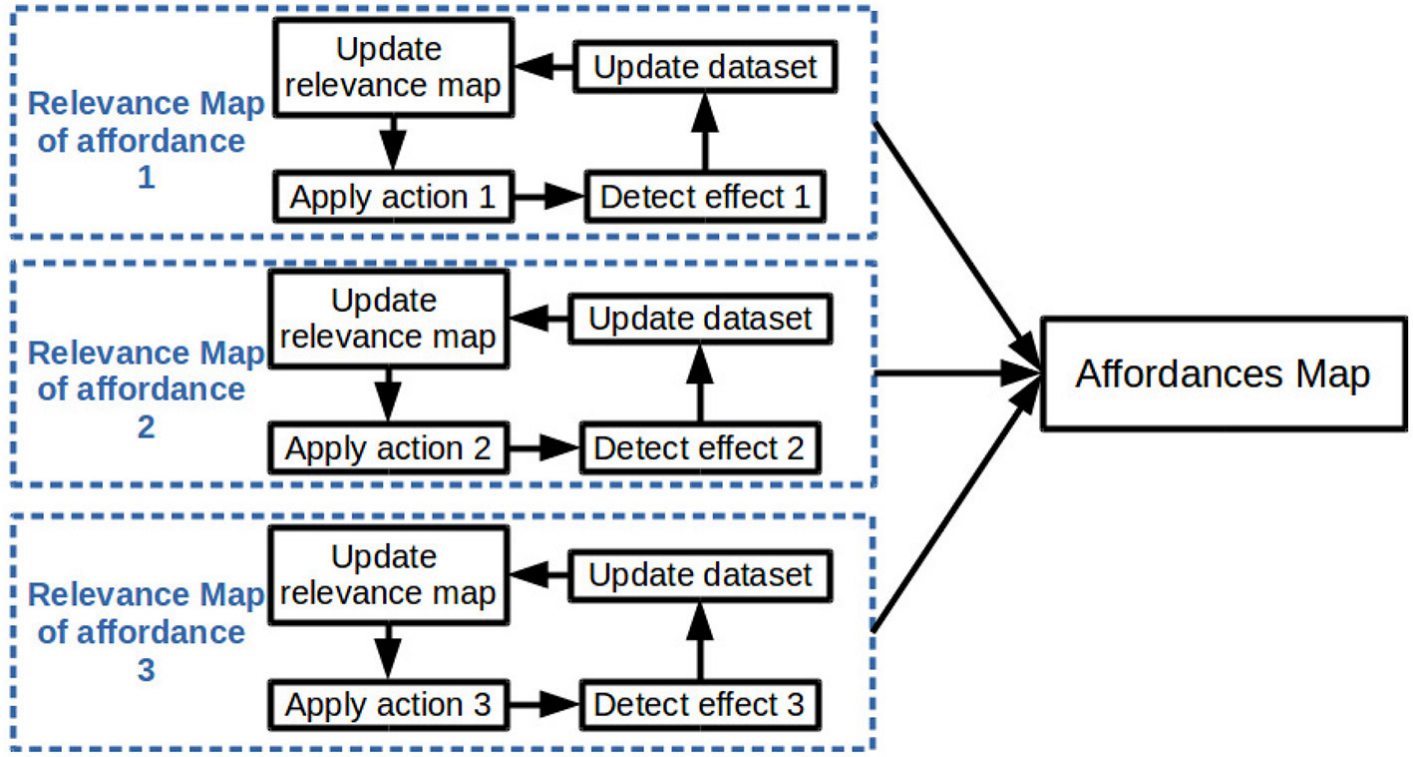
Overview of the general approach to build an affordances map.

The general approach, summarized in Figure 1, is to separately build the relevance map relative to each considered affordance. Each relevance map is built by collecting data through the interactions of the robot and then by training online a classifier on the data. The classifier is used to build the relevance map by attributing weights to segments extracted from the current scene (see Sections 3.2.2 and 3.2.4). Finally each relevance map is merged in one affordances map (see Section 3.3).

A formalization of affordances is proposed in Section 3.1. Then, in Section 3.2, the workflow to build a relevance map is explained.

### 3.1. Affordances Formalization

In this study, an affordance is a relation ϕ between an action *a* and an effect *e*. This relation is formalized as a conditional probability of an effect *e* to occur after the application of an action *a* on an element with a visual feature *X* (see Equation 1). Thus, ϕ is a function parameterized by *a* and *e* which takes as input *X* and gives as output a value between 0 and 1. This value represents the probability of existence of the affordance which draws a relationship between the triplet *a*, *e*, and *X*.


(1) $$\begin{array}{l} \phi_{\left(a,e\right)}\left(X\right)=P\left(\Delta =\left(a,e\right)|X\right) \end{array}$$

In this equation, Δ denote the random variable which takes as value a pair of action and effect.

In this study, our method is tested with three affordances : *pushable* objects, *liftable* objects, and *pressable* buttons. The *pressable* and *pushable* are simple affordances which can be represented by Equation (1) with Δ = (*push, moved*) and Δ = (*press, activated*). Regarding the *liftable* affordance, we assume that a *liftable* object is surely *pushable*. To consider this case, based on the Equation (1), we define a *composite affordance* as an affordance based upon one or several affordances.


(2) $$\begin{array}{l} \;P\left(\Delta_{1}|X\right)=P\left(\Delta_{1}|X,\Delta_{0}\right)P\left(\Delta_{0}|X\right)\\ \phi_{\left(a_{1},e_{1}\right)}\left(X\right)=P\left(\Delta_{1}=\left(a_{1},e_{1}\right)|X,\Delta_{0}=\left(a_{0},e_{0}\right)\right)\phi_{\left(a_{0},e_{0}\right)}\left(X\right) \end{array}$$

Equation (2) presents the formal representation of a composite affordance which links an action *a*_1_ and an effect *e*_1_ to an action *a*_0_ and an effect *e*_0_. It assumed that if the feature *X* affords the action *a*_1_ by producing the effect *e*_1_ then it affords the action *a*_0_ by producing the effect *e*_0_ too. In other words, the existence of the affordance associated with Δ_1_ = (*a*_1_, *e*_1_) implies the existence of the affordance associated with Δ_0_ = (*a*_0_, *e*_0_). In the following text, we say that the probability of *X* to afford *a*_1_ by producing the effect *e*_1_ is *filtered* by the probability of *X* to afford *a*_0_ by producing the effect *e*_0_. The Equation (2) is obtained by the Bayes' rule. The proof is presented in Appendix A.

Equation (3) presents the general case of a composite affordance as a composition of several other affordances. For this equation to be true, all the component affordances must be independent from each other.


(3) $$\begin{array}{l} \;\phi_{\left(a,e\right)}(X)=\\ P(\Delta =(a,e)|X,\overset{n}{\underset{i=0}{\cap }}\Delta_{i}=(a_{i},e_{i}))\prod_{i=0}^{n}\phi_{\left(a_{i},e_{i}\right)}(X) \end{array}$$

For instance, in this article, the probability of something to be *liftable* is filtered by the probability of something to be *pushable*. Because we assume that something *liftable* is also *pushable*, thus the *liftable* affordance requires the *pushable* affordance.

### 3.2. Workflow to Build a Relevance Map

#### 3.2.1. Overview

Our method aims at building an affordances map through an autonomous exploration driven by a robot equipped with two arms. The affordances map is the combination of several relevance maps. Each of them is relative to a specific affordance. To build one relevance map, the robot explores the environment which is unknown, with a specific action primitive. An action primitive is a pre-programmed sequence of motor commands to achieve a specific action. The system detects a possible effect thanks to a process, named here effect detector, detecting the changes in the environment produced by an action primitive. Thanks to the interaction and the effect detector, labeled samples are collected. They are labeled with a value of 1 if the interaction produced an effect, with a value of 0 otherwise. A classifier is used to build the relevance map. It is trained online in order to use the relevance map to drive the exploration. The visual system of the robot is an RGB-D camera (Microsoft Kinect v2) which generates 3D pointclouds. The Kinect v2 offers several resolutions, in this work, we use a resolution of 960 × 540 which offers a compromise between performance and quality. Thus, the 3D pointclouds are composed of 518,400 points. The action primitives and effect detector used in this study are described in Section 3.2.6.

**Figure 2:**
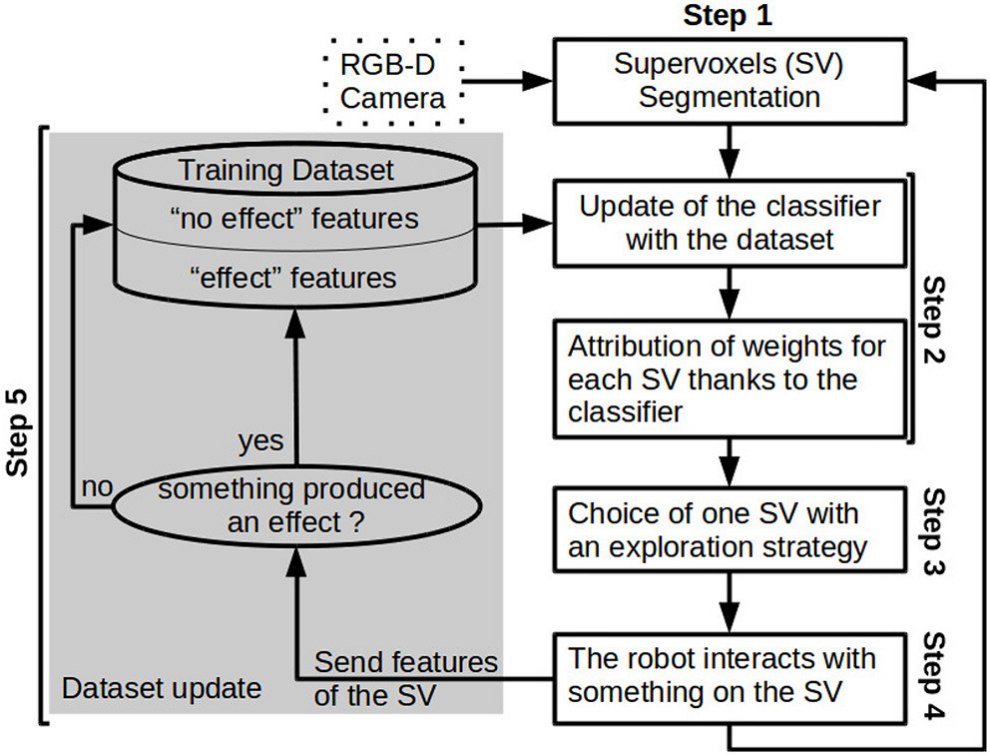
Overview of the workflow followed during an exploration to build a relevance map.

The exploration is sequential, the robot interacts with the environment, observes the effect, updates its perception and starts again. During the interaction, the system does not update its perception. The workflow of one iteration (shown in Figure 2) follows 5 steps:

- **Step 1:** An oversegmentation of the 3D pointcloud into *supervoxels* using Voxel Cloud Connectivity Segmentation (VCCS) method is done on the current scene. Visual features are extracted from each supervoxels. Supervoxel segmentation is described in Section 3.2.2 and the visual feature extraction method is explained in Section 3.2.3.
- **Step 2:** The classifier is updated with the training dataset extended with a new labeled sample. Then, the classifier weights are attributed to each supervoxel. The outcome is the relevance map of the current scene. This step is explained in Section 3.2.4.
- **Step 3:** The next supervoxel to interact with is chosen as a target for the action primitive. Section 3.2.5 explains how the target is chosen.
- **Step 4:** An action primitive is applied on the center of the chosen supervoxel. Each action primitive is explained in Section 3.2.6.
- **Step 5:** To check if an effect is produced by the action primitive, an effect detector is applied. The visual feature of the selected supervoxel is added to the training dataset with a label indicating if there was an effect. The different effect detectors are described in Section 3.2.6.

#### 3.2.2. Supervoxels

The relevance map relies on supervoxels segmentation. Supervoxels were introduced by Papon et al. (2013a) with his voxel cloud connectivity segmentation (VCCS) method. A supervoxel is similar to a superpixel like in SLIC (Achanta et al., 2010) or turbopixel (Levinshtein et al., 2009) methods except that it integrates depth information. Contrary to superpixel segmentation, VCCS works directly on a 3D pointcloud. A supervoxel is a cluster of voxels. A voxel is the smallest unit in a 3D image. In a pointcloud, a voxel is a point. The use of depth information allows the supervoxels to respect the boundary of objects which is a significant enhancement compared with superpixels. So, the information extracted from a supervoxel is more likely to be relative to a single component of the environment. Thus, this information is more consistent.

VCCS method workflow is the following: voxel seeds are evenly distributed on the pointcloud, then with local nearest neighbor, regions grow from these seeds by adding voxels. The neighborhood is defined by a radius named seed radius (*R*_*seed*_). This hyperparameter controls the size of the supervoxels. The local nearest neighbor uses a distance (see Equation 4) composed of CIELab^1^ color distance (*D*_*c*_), spatial distance (*D*_*s*_), and shape distance (*D*_*f*_) computed by the fast point feature histogram (FPFH, Rusu et al., 2009) algorithm.


(4) $$\begin{array}{l} D=\sqrt{\frac{\lambda D_{c}^{2}}{m^{2}}+\frac{\mu D_{s}^{2}}{3R_{seed}^{2}}+\epsilon D_{f}^{2}} \end{array}$$

As shown in Equation (4), three weights λ, μ, and ϵ control the importance of each distance. Therefore, VCCS algorithm has four important hyperparameters. Also, *m* is a constant to normalize the distance in the CIELab space. *R*_*seed*_ controls the size of the supervoxels and (λ, μ, ϵ) control their shapes. Only the size of the supervoxel is critical because if an object is smaller than a supervoxel then the information extracted from it will not be consistent. While, for the three other parameters, they can be tuned to have meaningful supervoxels for a large range of environments. The values used in this study for these parameters are listed in Table 1.

**Table 1:** Value of the hyperparameters of VCCS used for the experiments in the present paper.

***R*_*seed*_**	**λ**	**μ**	**ϵ**
0.05	0.2	0.4	0.4

A major drawback of VCCS is the inconsistency of the segmentation over a video stream. When extracted on a video stream, the segmentation is different for each frame even if the scene is static. This due to the noise of the depth image.

In this work, supervoxels are used as the smallest visual unit for image processing as well as for the action primitives targets. The version used is the VCCS implemented in the PointCloud Library (Rusu and Cousins, 2011). In this implementation, the algorithm gives as output a centroid point for each supervoxels which is at the average position, has the average color and normal of the points in the supervoxel. Also, an adjacency map is provided which represents a graph of Euclidean proximity of each supervoxel. Therefore, to find the neighbors of a supervoxel, going through the adjacency map is enough.

#### 3.2.3. Features Extraction

The visual features extracted from the supervoxels and used to train the classifier are the concatenation of color histograms with the CIELab encoding and a geometric descriptor based on FPFH.

For each channel of the CIELab color, a five-bin histogram is extracted. Then, they are concatenated into one vector of 15 entries.

Fast point feature histogram (FPFH) proposed by Rusu et al. (2009) is a widely used geometrical descriptor. It is appreciated for its high discriminative capacity. In the present method, FPFH is extracted on the central point of the pointcloud including the targeted supervoxel and its neighbors. The radius of neighborhood to compute FPFH is set to the size of a supervoxel, thus the central point FPFH takes into account the whole considered pointcloud. The central point is the centroid of the targeted supervoxel. This feature has 33 dimensions.

#### 3.2.4. Building a Relevance Map

Thanks to the online trained classifier, the supervoxels are weighted with values between 0 and 1. A weight represents the relevance of a supervoxel, i.e., the probability of a supervoxel to be part of a component which will produce an effect after the application of a certain action. Thus, a relevance map is a set of weighted supervoxels. The classifier called the Collaborative Mixture Models (CMMs) was introduced in our previous work (Le Goff et al., 2019).

The conditional probability which formalized an affordance is the output of CMMs. CMMs are used to classify samples between two classes (*a, e*) and $\left(a,\bar{e}\right)$. The first one is the class of *effect occurrence* and the second one is the class of *absence of effect* after the application of action *a*. Equation (5) defines the probability of a feature *X* to be part of the class (*a, e*).


(5) $$\begin{array}{l} P\left(\Delta =\left(a,e\right)|W,\Theta ,X\right)=\frac{1+\Gamma \left(W_{e},\Theta_{e},X\right)}{2+\Gamma \left(W_{e},\Theta_{e},X\right)+\Gamma \left(W_{\bar{e}},\Theta_{\bar{e}},X\right)} \end{array}$$

Where Γ is a Gaussian mixture model (GMM), *W*_*e*_ are the weights associated to the GMM of class (*a, e*), Θ_*e*_ are the parameters of the multivariate normal distributions of the GMM associated to (*a, e*), $W=W_{e}\cup W_{\bar{e}}$, and $\Theta =\Theta_{e}\cup \Theta_{\bar{e}}$. 1 is added to the numerator and 2 is added to the denominator to obtain a default probability of $\frac{1}{2}$ if both mixtures are empty.

The parameters of CMMs are the following:

- *K*_*E*_: number of components of the mixture models encoding the class (*a, E*) with $E\in \left\{e,\bar{e}\right\}$. This number is estimated during the training.
- *S* = {_*s*_*i*_, Δ_*i*_}*i*<*I*_: database of samples and their corresponding label constituted during the exploration.
- Θ_*E*_ = {_μ_*k*_, Σ_*k*_}*k*<*K*_*E*__: parameters of the multivariate normal distribution of model associated to class Δ with mean μ_*k*_ and covariance matrix Σ_*k*_. They are estimated by their sample estimator.
- *W*_*E*_ = {_*w*_*k*_}*k*<*K*_*E*__: weights of the mixture model associated with class (*a, E*). These parameters are computed thanks to their sample estimators.
- $\Delta \in \left\{\left(a,e\right),\left(a,\bar{e}\right)\right\}$: class to be predicted by the classifier.

Each class is modeled by a GMM and each GMM is composed of several multivariate normal distributions. A distribution models a component. The means and the covariances of the distributions are computed by their samples estimators.

**Figure 3:**
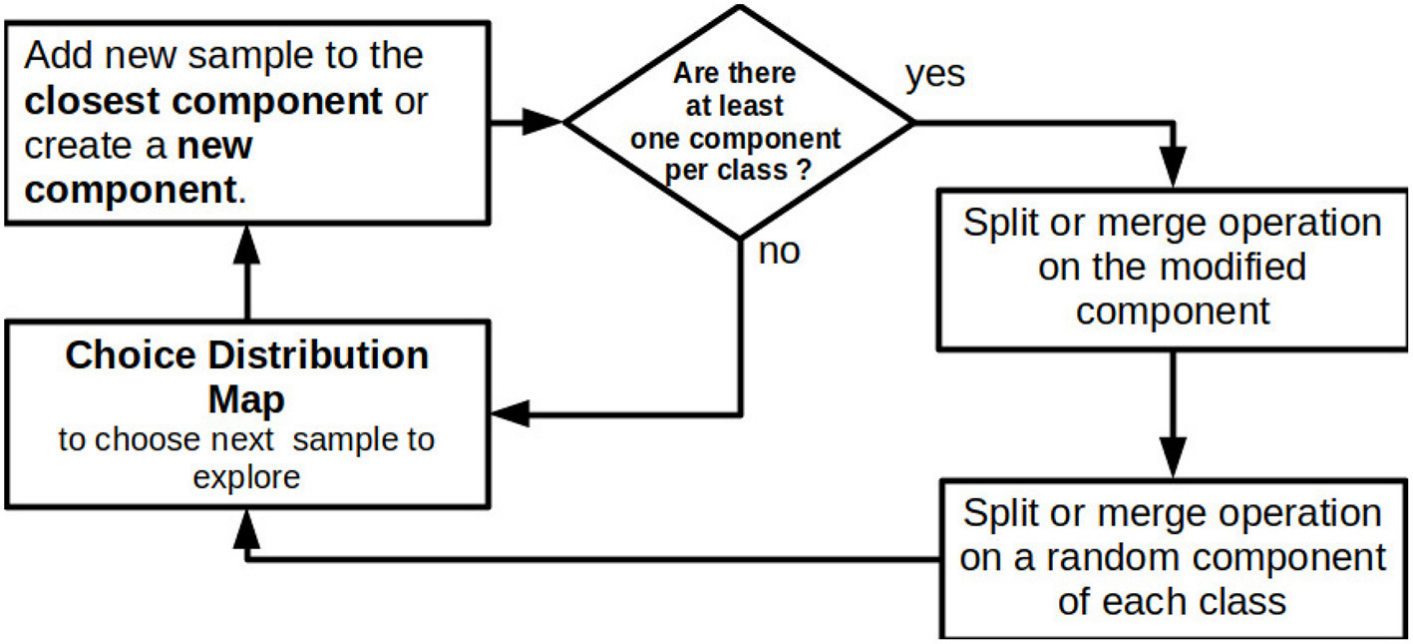
Schema of the training algorithm of CMMs.

Figure 3 summarizes the training algorithm used to build the models. CMMs are trained online in iterations in which a single new sample is added at a time, along with its label. Adding a sample consists of three main steps:

1. If there is no component yet in the class of the new sample, create a component with as mean the sample and an initial fixed covariance, otherwise, find the closest component and add the sample to this component. Finally, update the parameters of the component;
2. A *split* operation is applied to the updated component. If it is not successful the *merge* operation is then applied on the same component;
3. One component per class is randomly chosen and the *split* operation is applied to each of them. If the selected component is not split then the *merge* operation is applied on it.

From the previous algorithm introduced in Le Goff et al. (2019), some minor changes have being made. They are described in Appendix B.

#### 3.2.5. Choice of the Next Area to Explore

From the predictions of the classifier, a choice distribution map is computed. The choice distribution map is also a set of weighted supervoxels, but a weight represents the probability for the supervoxel to be chosen by the system as the next target of the interaction. A weight is the combination of the *uncertainty* and the *confidence* of the classifier. The higher the uncertainty and the lower the confidence, the higher is the probability for a supervoxel to be chosen.

Uncertainty are computed together in one metrics and confidence in another. Then they are combined to output a probability of choice of a feature *X*_*i*_ of the *i*^*th*^ supervoxel extracted on a pointcloud as shown in Equation (6).


(6) $$\begin{array}{l} P_{c}\left(X_{i}\right)=u\left(X_{i}\right)*\left(1-c\left(X_{i}\right)\right) \end{array}$$

Where *u*(.) is the uncertainty and *c*(.) is the confidence.

##### Uncertainty

As CMMs is a probabilistic classifier, its output can give directly an uncertainty measure. The output of CMMs is a probability of membership of a sample in a class (see Equation 5). The closer this probability to 0.5, the more uncertain the classification is. Uncertainty of classification is computed thanks to the Equations (7) and (8).


(7) $$\begin{array}{l} u\left(X_{i}\right)=\{\begin{array}{cc} f\left(p\right) & \left|S_{e}|<=|S_{\bar{e}}\right|\\ f\left(1-p\right) & \left|S_{e}|>|S_{\bar{e}}\right| \end{array} \end{array}$$

where *p* = *P*(Δ = (*a, e*)|*W*, Θ, *X*), *S*_*e*_ is the set of samples for effect *e* and $S_{\bar{e}}$ the set of samples for no effect and *f* is the following function:


(8) $$\begin{array}{l} f\left(x\right)=\{\begin{array}{cc} -2x\left(log\left(2x\right)-1\right) & x>=0.5\\ -4x^{2}\left(log\left(4x^{2}\right)-1\right) & x<0.5 \end{array} \end{array}$$

**Figure 4:**
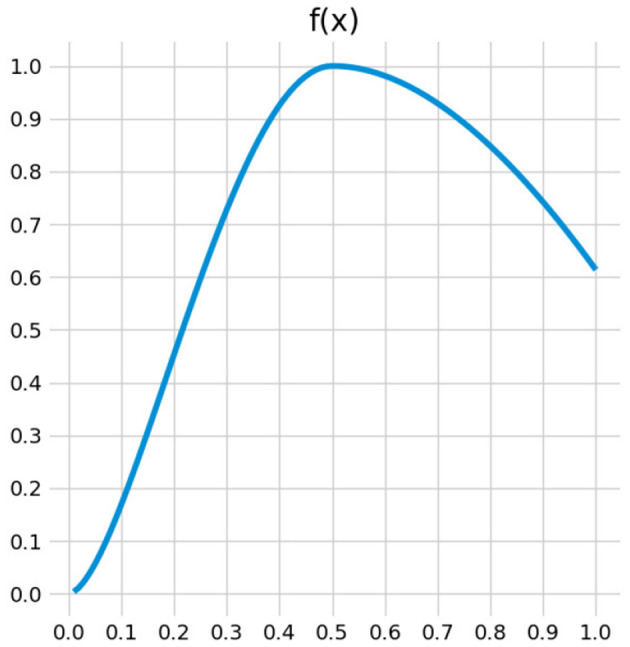
Function used for uncertainty estimation (*f*). This function gives a higher probability of choice to uncertain classification, but also to certain classification to the chosen class, i.e., the one with fewest samples.

The function *f*(.) is plotted in Figure 4.

The function of Equation (8) and represented in Figure 4 gives a higher value for classification equal or greater to 0.5. Therefore, the uncertainty computed this way drives the exploration to collect samples with features from uncertain area but also from areas predicted as part of the class with the less samples collected yet. Thus, the exploration process tends to balance the dataset between the classes as defined in Equation (7).

##### Confidence

The classification of CMMs relies on a mapping of the feature space of normal distributions. The border of these distributions can give an insight on the least dense areas in the feature space. The confidence of the classifier for a sample is its probability of membership in its closest component. This probability is computed thanks to Equation (9).


(9) $$\begin{array}{l} P\left(K_{E}=k|X,\Theta ,\Delta \right)=\frac{w_{k}*G\left(\mu_{k},\Sigma_{k},X\right)}{\sum_{i=0}^{K_{E}-1}w_{i}*G\left(\mu_{i},\Sigma_{i},X\right)} \end{array}$$

By choosing areas with the lowest confidence, the exploration gives a focus to areas in which the system has less information. Therefore, this metric could be interpreted as an approximation of entropy.

#### 3.2.6. Action Primitives and Effect Detectors

##### Pushable Objects

The pushable affordance is associated to a push primitive and a change detector as effect detector. The push primitive is going through three steps. First, the end-effector is going in an approach pose near and oriented toward the target. Then, the end-effector follows a straight line toward the target until going through it. Thus, if a pushable object is on the target, it will be pushed. Finally, a reverse motion is applied in which the arm goes back to its home position. For each interaction, the left or the right arm is randomly chosen. If no valid plan is found with the chosen arm then planning is tried with the second arm.

The planning is done within the MoveIt framework (Şucan et al., 2012; Şucan and Chitta, 2019). This framework provides planning algorithms with obstacle avoidance. Obstacle avoidance is used during the approach motion to prevent any involuntary disturbance in the scene.

**Figure 5:**
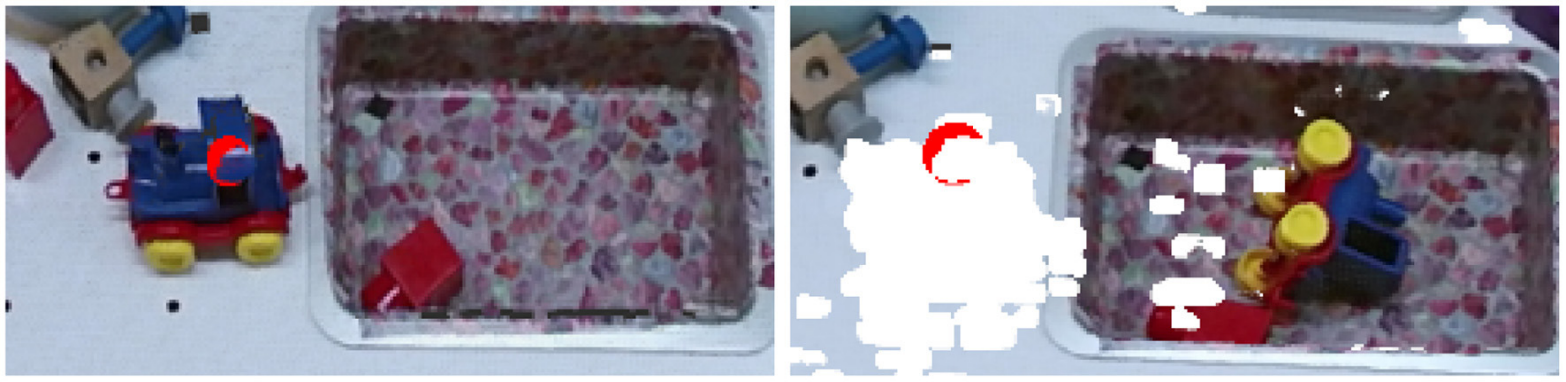
Visualization of the change detector. The right picture represents a part of a scene before a push and the left picture after a push. The red dot on both pictures represents the target of the push primitive which is here the upper part of the blue toy. This target corresponds to the center of a supervoxel. The white areas represent the parts detected as different between both images.

The effect detector is a simple change detector of the scene. As shown in the Figure 5, a difference point cloud is computed (in white in the Figure 5) by substracting the pointcloud before (the left picture of the Figure 5) and the one after the interaction (the right picture of the Figure 5). Then, if the points of the targeted supervoxel (its center is represented by a red dot in the Figure 5) is part of the difference pointcloud then a change has occurred.

##### Pressable Buttons

**Figure 6:**
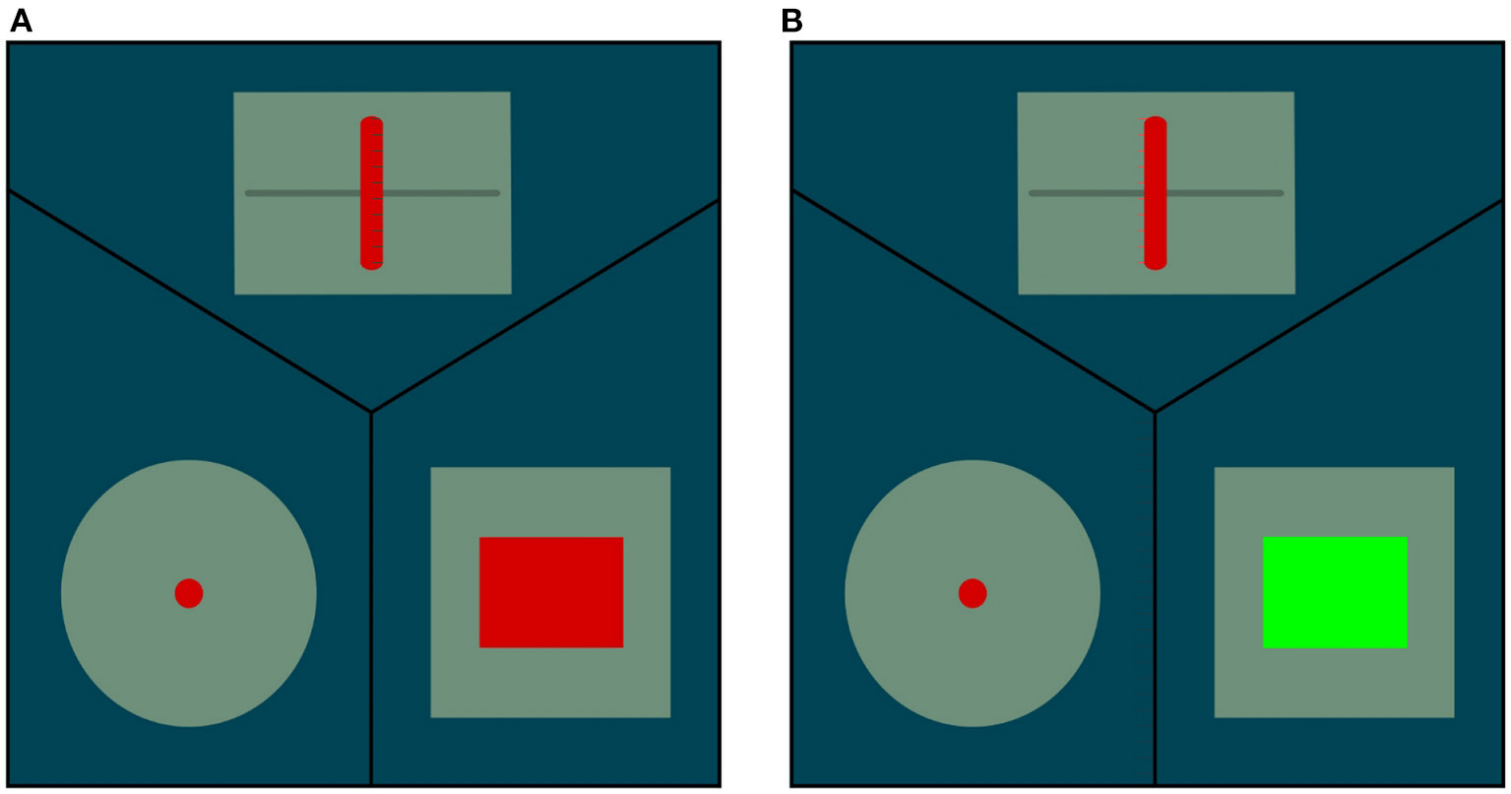
Interface which displays on a screen the state of different interactive modules. For the present study, only the right bottom part is used. It displays the buttons' state. The rectangle is red if no button is pushed and it becomes green if at least one button is pushed. **(A)** No button pushed. **(B)** At least one button is pushed.

This affordance is associated with pressable buttons which activate a signal displayed on a screen visible to the robot. The action primitive is similar to the push primitive except for the orientation which is only vertical or horizontal in the robot frame (the push primitive used to learn the pushable affordance has a continuous range of orientations). The effect detector is a recognition system which allows the robot to see if a button is pushed. The state of the buttons is displayed on a screen like in the pictures of Figure 6. The state is perceived by the robot through a visual recognition system implemented with OpenCV. This system is specific to the interface.

##### Liftable Objects

Among the pushable parts in the environment the robot will try to learn liftable parts. It is assumed that liftable parts are first pushable, thus, liftable affordance is a composite affordance, composed by the pushable affordance. The probability to afford the lift primitive is filtered by an already learned probability to afford the push primitive. Therefore, the exploration is biased by a relevance map of pushable affordance.

For this affordance, the robot uses a lift primitive that consists in going above the target, rotating the wrist of its gripper in a certain orientation, then going down and closing the gripper before finally going up again and letting the lifted “thing” fall by reopening the gripper. Like for the push primitive during the approach motion to go above the target, the obstacles are avoided.

To detect if something is lifted, the opening of the gripper is checked before reopening the gripper. If it is not fully closed, the target will be considered as lifted.

In this primitive the gripper is fixed in the vertical orientation, thus, only liftable objects laying on a horizontal plane are considered here. The approach can be extended to any liftable object with an appropriate lift primitive.

### 3.3. Building the Affordances Map

The affordances map is a combination of several relevance maps. Each supervoxel has a set of weights assigned corresponding to each relevance. All the weights under a certain threshold are reduced to zero. The affordances map is represented by assigning a color to each affordance and no color for supervoxels with weights all equal to zero.

## 4. Experiments

### 4.1. Protocol

For each of the three affordances, 4 experiments have been conducted. An initialization step has been added in the experiments of liftable objects and pressable buttons in which the system is forced to gather at least 10 samples of each class. With a uniform random sampling, the chance to gather positive samples in these experiments is very low, thus at the beginning of the experiment, the robot collects only negative samples. This initial step allows the system to start from a balanced dataset. Adding this step was not useful for the experiments with the push affordance as the probability to gather positive samples is higher.

**Figure 7:**
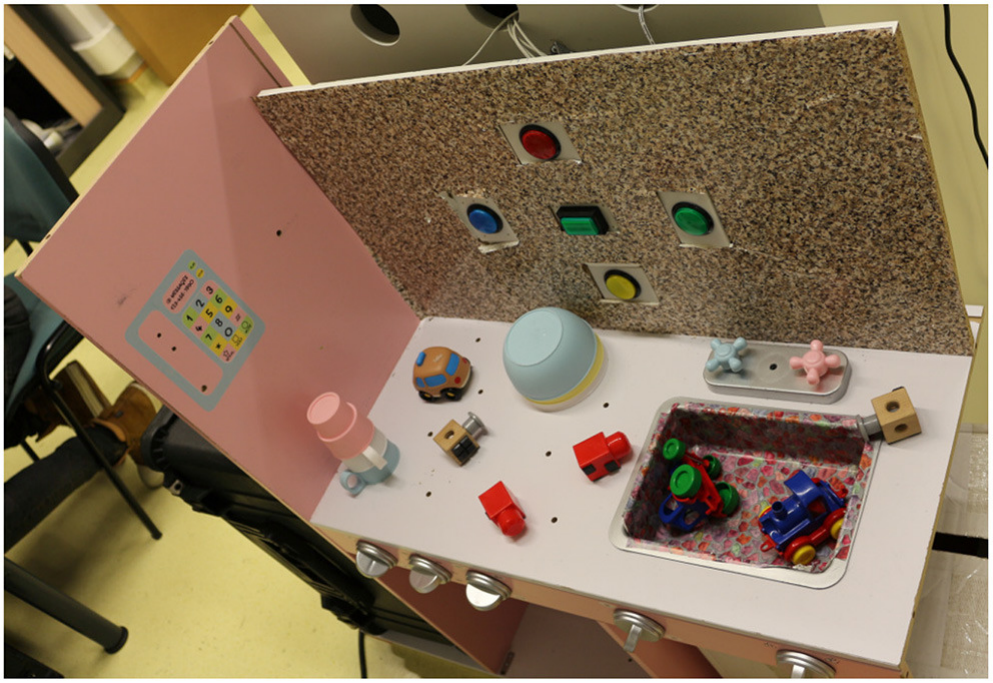
The setup used for all the experiments. The setup is a toy kitchen with 5 interactive push buttons integrated into a vertical plane.

**Figure 8:**
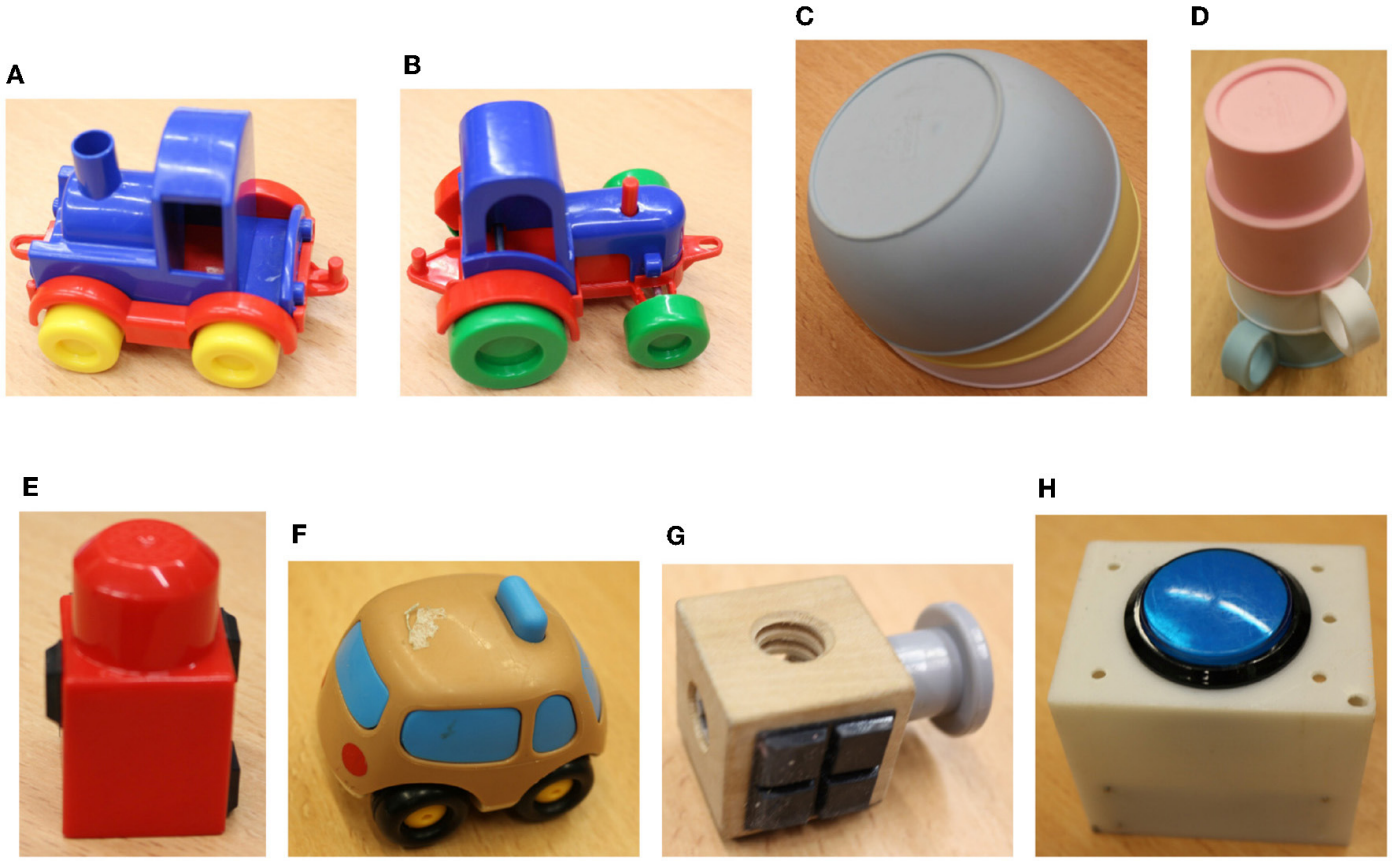
Eight different types of objects used in the experiments. The affordance expected to be linked to these objects is indicated in bold. **(A)** Toy locomotive: **pushable, liftable**. **(B)** Toy locomotive: **pushable, liftable**. **(C)** Pile of bowls: **pushable**. **(D)** Pile of mugs: **pushable**. **(E)** Duplo bricks: **pushable, liftable**. **(F)** Toy car: **pushable**. **(G)** Wooden cube: **pushable, liftable**. **(H)** Buttons: **pressable**.

Figure 8 is a collection of pictures representing the objects used in the experiments: 3 bowls in a pile, 3 mugs in a pile, two different toy locomotives, Duplo bricks, two identical wooden cubes, and 5 buttons. Of course, the pile of bowls and mugs (see Figures 8C,D) can be dismantled during an experiment. The Duplo bricks are of different colors (red in the Figure 8E): green, red, purple, orange, yellow. There are five buttons, all are visible in Figure 7: circular blue (the one in Figure 8H), red, yellow, green and squared green. Figure 8 indicates in bold for each object its expected affordance.

### 4.2. Quality Measures

To assess the performance of the trained classifier precision, recall, and accuracy are computed by following the Equations (10) and (11). These measures are computed according to a ground truth. The ground truth is obtained from a snapshot of the scene without the objects that afford the studied action which corresponds to the background. For the pushable affordance, the ground truth is exact as it corresponds just to the background and the buttons. For the pressable buttons, the ground truth is approximative because only a part of the button is pressable, the colored central part (see 8h) while the ground truth we have set takes into account the whole white box. Thus, the performances should be slightly better than the one presented in the section 5. For the liftable affordance, the ground truth is even less accurate as it corresponds to our a priori about what the robot may lift or not. An autonomous exploration is interesting and useful precisely when the ground truth is difficult to set. In our case, it is difficult to predict exactly the robustness of the lift according to the robot capacity and the designed lift primitive.


(10) $$\begin{array}{l} precision=\frac{tp}{tp+fp}\\ \;recall=\frac{tp}{tp+fn}\\ accuracy=\frac{1}{2}\left(\frac{tp}{GT_{e}}+\frac{tn}{GT_{\bar{e}}}\right) \end{array}$$

Where *tp* is the number of true positives and *tn* is the number of true negatives [i.e., supervoxels well-classified in the class (*a, e*) and $\left(a,\bar{e}\right)$]; *fp* are false positives, i.e., supervoxels misclassify as part of class (*a, e*) and *fn* are false negatives, i.e., supervoxels misclassified as part of class $\left(a,\bar{e}\right)$; and *GT*_*e*_ is the ground truth for parts of the environment that produced the expected effect and $GT_{\bar{e}}$ is the ground truth for parts of the environment that do not produce the expected effect. Their definitions, for N supervoxels extracted from a pointcloud, is the following:


(11) $$\begin{array}{l} tp=\overset{N}{\underset{i}{\sum }}P\left(\Delta =\left(a,e\right)|W,\Theta ,x_{i}\right)*\left(1-\delta_{i}\right)\\ tn=\overset{N}{\underset{i}{\sum }}P\left(\Delta =\left(a,\bar{e}\right)|W,\Theta ,x_{i}\right)*\delta_{i}\\ fp=\overset{N}{\underset{i}{\sum }}P\left(\Delta =\left(a,e\right)|W,\Theta ,x_{i}\right)*\delta_{i}\\ fn=\overset{N}{\underset{i}{\sum }}P\left(\Delta =\left(a,\bar{e}\right)|W,\Theta ,x_{i}\right)*\left(1-\delta_{i}\right)\\ GT_{e}=\overset{N}{\underset{i}{\sum }}1-\delta_{i}\\ GT_{\bar{e}}=\overset{N}{\underset{i}{\sum }}\delta_{i} \end{array}$$

Where δ_*i*_ is the Kronecker symbol equal to 1 if the i^*th*^ supervoxel is part of the background, and otherwise equal to 0; *x*_*i*_ represents the features of the i^*th*^ supervoxel.

These measures are widely used as quality measures for supervised learning algorithm.

## 5. Results

For each experiment, the precision, recall, and accuracy scores of each replication are presented separately to avoid losing information.

**Figure 9:**
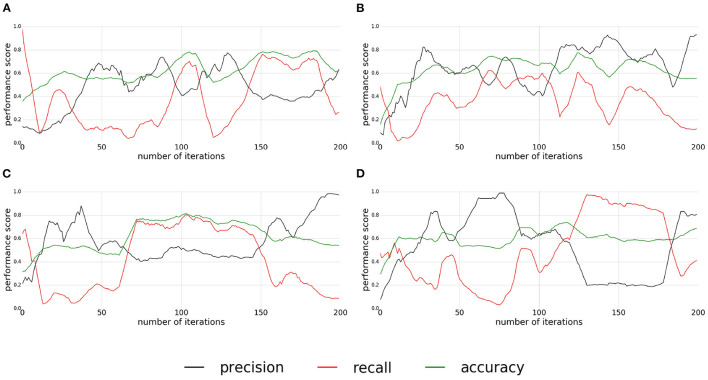
**(A–D)** Plots of precision, recall, and accuracy for pushable affordance. Each plot correspond to one replication of the experiment.

The precision, recall, and accuracy scores of the experiment for the pushable affordance (presented in Figure 9) are satisfying considering the complexity of the setup. The classification quality is very different for each replication. In the first experiment (Figure 9A), the classifier converges only around the 150th interaction with an accuracy around 0.8, a recall varying between 0.6 and 0.8, and a low precision around 0.4. Finally, for this replication, the quality drops at the end. For the second and third experiments (Figures 9B,C) the classifier converges around the 60th interaction. For the second replication, the accuracy, recall, and precision are not stable and the classifier starts diverging after the 100th interaction. The classifier, of the third replication (Figure 9C), converges to an accuracy and a recall around 0.8 and a precision between 0.4 and 0.5 and stays stable. But it diverges after the 150th interactions. For the last replication (Figure 9D), it is difficult to isolate a period of convergence of the classifier. The classification quality of this experiment is very unstable.

**Figure 10:**
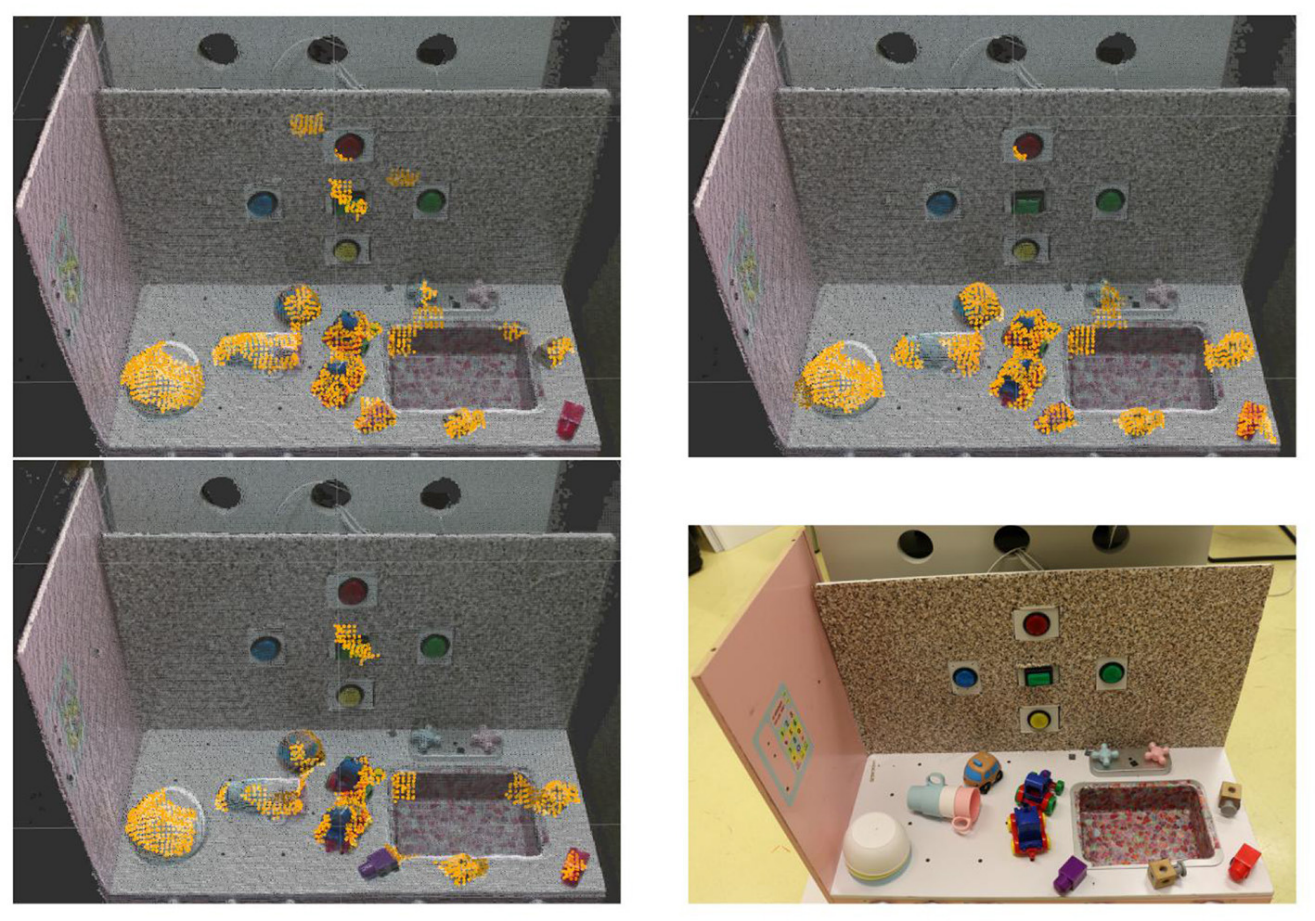
Three push relevance maps extracted from the same scene and with the same classifier on a video stream. The differences between the three maps is due to the extraction of the supervoxels which produces a different segmentation at each frame. The bottom right picture represents the environment from which the relevance maps have been extracted.

For all the replications, the quality of classification diverges at the end. The divergence is probably due to mislabeled samples. The instability of the classification quality, clearly visible in the second replications, is due to the inconsistency of the supervoxel segmentation when extracted on a video stream as shown in Figure 10.

The Figure 10 shows three pictures representing push relevance maps. These relevance maps have been extracted with the same classifier on the same static scene on a video stream. The variability of the relevance map over these three images are due to the extraction of the supervoxels which produces a different segmentation at each frame. The variability of the segmentation is due to the noise of the depth stream. The higher the noise, the higher the variability is. On these pictures, the toy locomotives and the button are the noisiest areas. On these areas, the geometrical features can change a lot, which is due to the variation in the shape of the supervoxels.

The unstability of the results are more deeply discussed in Section 6.

**Figure 11:**
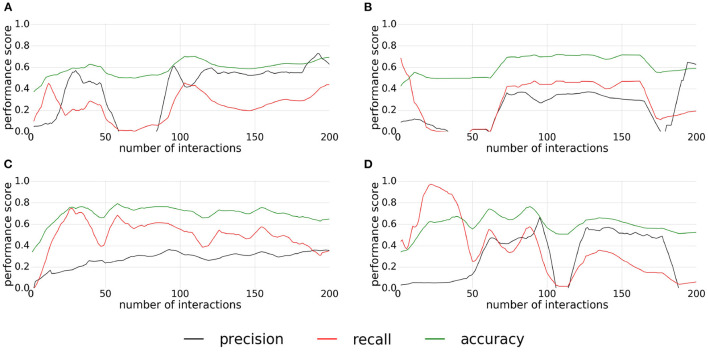
**(A–D)** Plots of precision, recall, and accuracy for pressable buttons. Each plot correspond to one replication of the experiment.

The precision, recall, and accuracy scores of the experiment with the buttons are shown in the Figure 11. In this experiment, the replications give also different results. For the first replication (Figure 11A), the classifier converges around the 80th interaction and keep the quality of classification steady around a value of 0.6 for the accuracy and the precision, a value of 0.5 for the recall. For the second replication (Figure 11B), the classifier converges around the 75th interactions with an accuracy around 0.7, a recall around 0.5 and a precision under 0.4, but this replication starts to diverge around the 160th interaction. For the third replication (Figure 11C), the classifier converges quickly to a value between 0.7 and 0.8 for the accuracy, around 0.6 for the recall while the precision increases slowly during all the replication. The accuracy and the recall slowly decrease after the 100th interaction. Finally, the last replication (Figure 11D) presents poor results. The classifier converges first between the 50th and 100th interaction, then diverges, and then converges again to a low classification quality, before finally diverging.

Overall, the classification is more stable for this experiment than for the experiments with the pushable affordance. The main difficulty in this experiment is that the buttons represent a small area. The size of the actual pushable area is even smaller, about the size of a supervoxel. This introduces noise on the extracted features. A solution may be to reduce the size of the supervoxels, but if a supervoxel contains too few points, the features could be inconsistent. Moreover, this reduced size creates a strong requirement in terms of the accuracy of the action primitive to prevent mislabeling.

**Figure 12:**
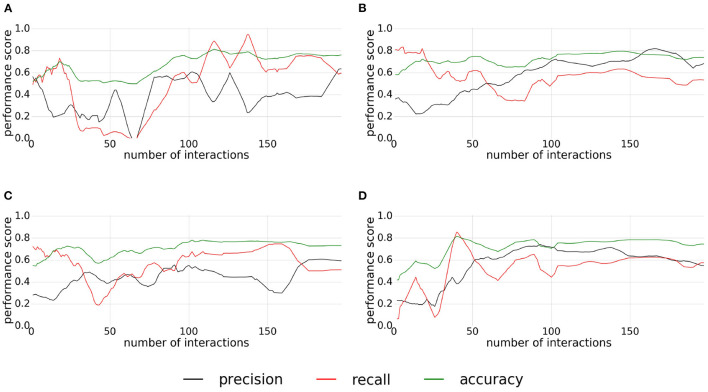
**(A–D)** Plots of precision, recall, and accuracy for liftable affordance. Each plot correspond to one replication of the experiment.

Figure 12 represents the performances monitored during the experiment conducted for the liftable affordances. For the first and the third replications (Figures 12A,C), the quality scores have similar shapes, the convergence is reached around the 100th interaction with a low precision and an accuracy, and a recall between 0.7 and 0.8. For the first replication, the recall, and precision are unstable between the 100th and the 150th interactions. In both, the recall and precision cross themselves to have a higher precision than recall which can be seen with a light decrease of the accuracy. For the second and fourth replications (Figures 12B,D), the classifier converges after the 100th interaction, with an accuracy around 0.8, a recall around 0.6, and a higher precision around 0.7. Unlike the two previous experiments (pushable objects and pressable buttons), the classification quality does not seem to diverge at the end of the experiment, except for the forth replication for which the precision decreases slowly after the 150th interaction.

As in the previous experiment, this experiment gives stable results. The low precision, observed on the first and third replications, is probably due to the inaccuracy of the ground truth. Finally, the stability of the convergence may be due to the use of a fixed push relevance map to filter the classification which does not change during the experiment.

**Figure 13:**
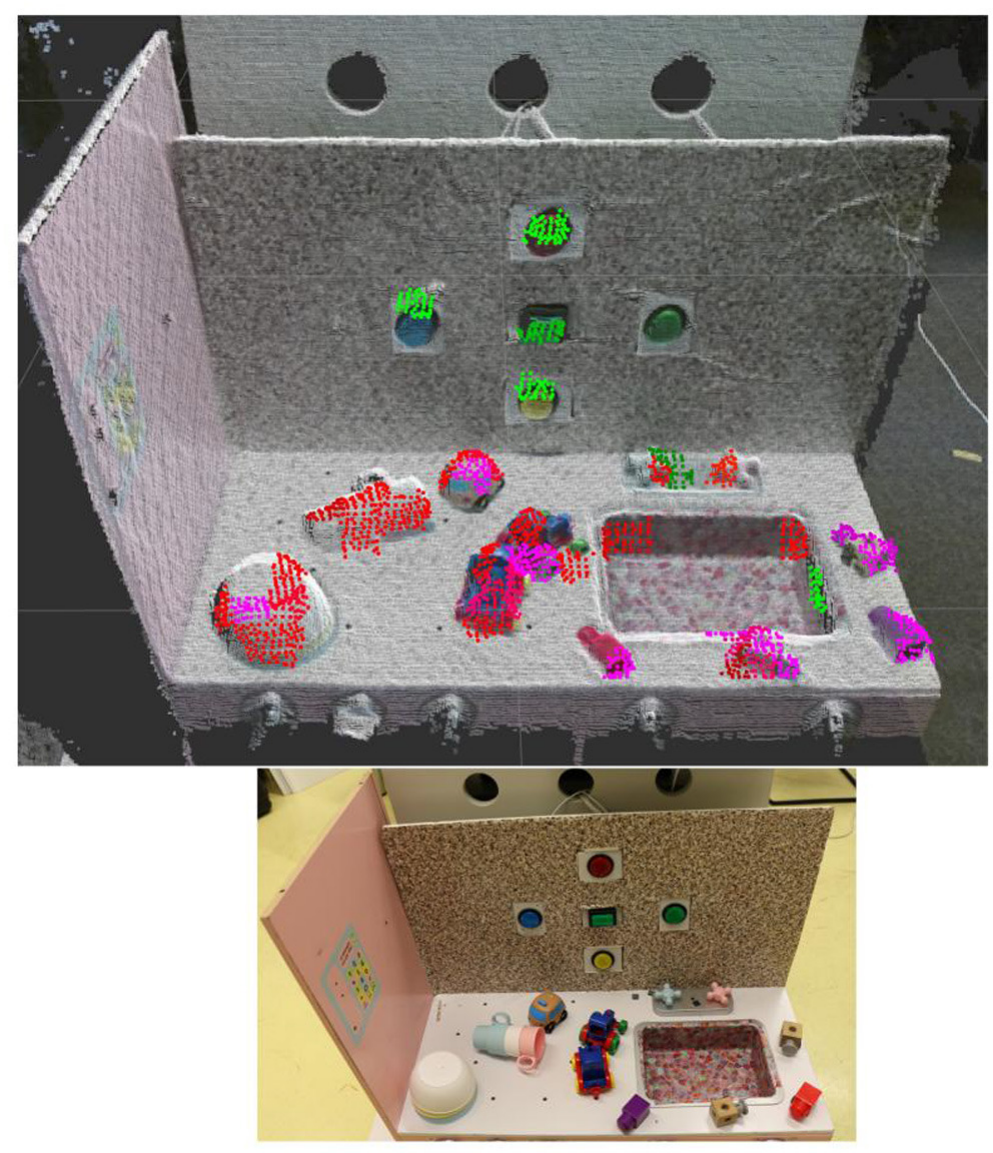
Affordances map of liftable pressable buttons and pushable affordances. Colored areas indicate areas classified with a probability above 0.5, in red to afford the push primitive, in purple to afford the lift primitive and in green to be an pressable buttons. The bottom picture represents the environment on which the affordances map has been extracted.

Figure 13 represents an affordances map obtained by the experiments described above. This map represents the areas categorized as pushable buttons in green, as liftable objects in purple, and as pushable objects in red. It was obtained by selecting the best performing classifier among the experiments and at the best moment inside a replication. Only supervoxels of both relevance maps with a probability equal or higher of 0.5 are displayed.

An interesting property in this affordance map, is the low overlap between the parts predicted to be pushable and to be a pressable button. Also, as expected, the pile of bowl is detected as only pushable. The other objects are predicted as pushable and liftable or partially liftable. This affordances map is a proof of concept of what can be obtained with the proposed approach. For each experiment, more replications are required for a better assessment of the method robustness. The instability of the classifier needs to be dealt with for this approach to be more reliable.

The source code use to produce this results can be found on github^2^.

## 6. Discussion and Future Work

The experiments described in this article provide a proof of concept of learning affordances from the local level in a real environment. The results show the possibility of building affordances map from local features associated to a given action with a given expected effect. Although the results have shown a large variability over the four replications done for each affordance, relevance maps have been produced and combined into a meaningful affordances map. The relevance maps of the pressable and of pushable affordances do not overlap, which shows the capacity of the classifier to learn different concepts. The classifier is also able to refine a concept as shown in the experiment with the liftable affordance. Four different kinds of problems have been identified as the main causes of the observed variability and instability.

The most important one is the inconsistency of the supervoxel extraction over a video stream, as shown in Figure 10. This issue is directly linked to the active depth camera (kinect v2) used which produces noise on the depth images. Better cameras would alleviate the problem. Another possibility would be to include algorithms able to extract persistent supervoxels. The persistency of the supervoxels would allow to collect more data during an experiment and to remove some of the effect classification errors (Papon et al., 2013b).

The second problem is related to the quality of actions primitives and associated effect detectors. They greatly condition what could be learned by the system. Designing general and accurate action primitives in complex environments is a challenge. In our experiments, the poor accuracy of the button pressing and object lifting primitives probably plays a significant role in the instability of the method. As the generated affordances map represents the ability of action primitives to generate expected effects on each part of the environment, the precision and success rate of those primitives is critical and impacts the generated affordances map. This is actually expected: if a part of the environment does not consistently allow to obtain a given effect, it is normal that the classifier does not reliably considers it as affording the corresponding action. Further work on creating more elaborate, more reliable action primitives is thus expected to significantly improve the precision and stability of the generated affordances maps. Also, in the principle of developmental robotic, it will be interesting to connect this method to other developmental steps which would learn adaptive and accurate motor skills as well as effect detectors (Kim and Sukhatme, 2014; Jegorova et al., 2018). Any improvement with respect to this problem would significantly improve the results, as shown in our previous work (Le Goff et al., 2019) in which experiments in simulation have been conducted in a simplified setup with no primitive and a “perfect” effect detector.

The third problem comes from the features taken into account by the classifier. Engineered descriptors have been used instead of learned ones. It has the advantage to require less data to train the system, but it comes at a price: the features may not be the most appropriate to distinguish the different parts of the environment. Relying on the data generated by the system would make the system more flexible and adaptive. To solve the data generation problem, the method could rely on engineered features at first and, once enough data have been generated, trained features could start to be taken into account by the classifier.

Finally, online learning brings the advantage of providing an estimation of environment parts category during exploration. It thus gives criteria to guide the exploration. However, the samples are processed in the order of arrival. At a certain point of the learning this order is optimized by the exploration process but at the beginning the order is random, following the underlying probability distribution of the explored environment. This initial phase is definitively a factor of the high variability of the method. A solution often used in online learning is a mechanism of unlearning (Cauwenberghs and Poggio, 2001; Bordes and Bottou, 2005; Saffari et al., 2009). An alternative would be to alternate between online and offline phases with batch learning.

## 7. Conclusion

In this study, a method is proposed to learn different perceptual maps called relevance maps relative to specific affordances. The framework is modular and thus permits to learn relevance maps relative to different affordances. In this article, as proof of concept, experiments have been conducted to learn relevance maps relative to pushable objects, liftable objects, and pressable buttons. Then, by combining these maps, a new perceptual map is obtained, called affordances map. This affordances map allows the robot to perceive the environment through its possible actions.

## Data Availability Statement

The raw data supporting the conclusions of this article will be made available by the authors, without undue reservation.

## Author Contributions

LKL is the main contributor. SD and AC have contributed equally for the writing and the scientific work. OY have contributed on the technical aspect. All authors contributed to the article and approved the submitted version.

## Funding

This work was supported by the DREAM project^3^ through the European Unions Horizon 2020 research and innovation program under grant agreement No 640891. This work has been partially sponsored by the French government research program Investissements d'avenir through the Robotex Equipment of Excellence (ANR-10-EQPX-44), and ANR-BMBF project Learn2Grasp (ANR-21-FAI1-0004).

## Conflict of Interest

The authors declare that the research was conducted in the absence of any commercial or financial relationships that could be construed as a potential conflict of interest.

## Publisher's Note

All claims expressed in this article are solely those of the authors and do not necessarily represent those of their affiliated organizations, or those of the publisher, the editors and the reviewers. Any product that may be evaluated in this article, or claim that may be made by its manufacturer, is not guaranteed or endorsed by the publisher.

## Footnotes

^1^CIELab is a colorimetry international standard from the International Commission on Illumination (CIE) of 1978.

^2^ https://github.com/robotsthatdream/wave1_relevance_map

^3^ http://www.robotsthatdream.eu

## Appendix A

This first appendix explains how the Equation 2, about affordances composition is obtained.

Let consider two affordances associated with Δ_0_ = (*a*_0_, *e*_0_) and Δ_1_ = (*a*_1_, *e*_1_). It is assumed that if the affordance associated with Δ_1_ exists for a visual feature *X* then the affordance associated with Δ_0_ exists for the same visual feature. In other words, if the action *a*_1_ produces an effect *e*_1_ when applied on a part of the environment with a visual feature *X* then it is certain that an effect *e*_0_ will occur after the application of an action *a*_0_ on the same part of the environment.

The Equation 2 is based on Bayes' rule :

(12)
$$\begin{array}{l} P\left(A|B\right)=\frac{P\left(B|A\right)P\left(A\right)}{P\left(B\right)} \end{array}$$

Lets start with the decomposition of the probability of existence of the affordance Δ_1_ knowing the action *a*_1_ was applied on a visual feature *X* and an affordance Δ_0_ exists with the Bayes' rule:

(13)
$$\begin{array}{l} P\left(\Delta_{1}|X,\Delta_{0}\right)=\frac{P\left(\Delta_{0}|X,\Delta_{1}\right)P\left(\Delta_{1}|X\right)}{P\left(\Delta_{0}|X\right)} \end{array}$$

We know that *P*(Δ_0_|*X*, Δ_1_) = 1 because, according to the assumption gave at the beginning of this appendix, if the probability that the affordance associated with Δ_1_ exists then there is a probability of one that the affordance associated with Δ_0_ exists. Therefore the equation become :

(14)
$$\begin{array}{r} P\left(\Delta_{1}|X,\Delta_{0}\right)=\frac{P\left(\Delta_{1}|X\right)}{P\left(\Delta_{0}|X\right)}\\ P\left(\Delta_{1}|X,\Delta_{0}\right)P\left(\Delta_{0}|X\right)=P\left(\Delta_{1}|X\right) \end{array}$$

## Appendix B

From the algorithm introduced in Le Goff et al. (2019), the *split* and *merge* have been modified. The maximization of the loglikelihood of the model to validate the splits and merges have been removed. Also, the split operation is limited to a maximum number of components per class. The Algorithms 1 and 2 are the new split and merge operations used in CMMs for the experiments in this article. The rest of the CMMs training algorithm remains unchanged.

**Algorithm 1:** MERGE algorithm

1: **procedure** MERGE(*C*,l,*M*_1_, ..., *M*_*N*_)
2: $C^{\prime }\leftarrow closest\text{\_}component\left(C\right)\in M_{l}$ ⊳ Search the closest component from *C* in *M*_*l*_
3: **if** *C*∩*C*′≠∅ **then** ⊳ If component C intersect with C'
4: $\tilde{C}\leftarrow C\cup C^{\prime }$
5: $M_{l}\leftarrow \left(M_{l}\backslash \;C,C^{\prime }\right)\cup \tilde{C}$
6: **end if**
7: **return** $M=\cup_{l=1}^{N}M_{l}$
8: **end procedure**

**Algorithm 2:** SPLIT algorithm

1: **procedure** SPLIT(*C*,l,*M*_1_, ..., *M*_*N*_)
2: **if** |*M*_*l*_| < *K*_*max*_ **then** ⊳ If the number of components of class *l* is above *K*_*max*_
3: **return** $M=\cup_{l=1}^{N}M_{l}$ ⊳ Then abandon the split
4: **end if**
5: $C^{\prime }\leftarrow closest\text{\_}component\left(C\right)\in M\backslash \left\{M_{l}\right\}$ ⊳ Search the closest component from *C* with a label ≠*l*
6: **if** *C*′∩*C*≠∅ **then** ⊳ If component C intersect with C'
7: *C*_1_, *C*_2_ = *split*(*C*)
8: *M*_*l*_←(*M*_*l*_\{*C*})∪{*C*_1_, *C*_2_}
9: **end if**
10: **return** $M=\cup_{l=1}^{N}M_{l}$
11: **end procedure**

*K*_*max*_ is the maximum number of components.
